# Spinal muscular atrophy with hypoplasia of the corpus callosum: a case report

**DOI:** 10.1186/s12883-023-03121-w

**Published:** 2023-02-18

**Authors:** Xiaomei Zhu, Hui Li, Chaoping Hu, Min Wu, Shuizhen Zhou, Yi Wang, Wenhui Li

**Affiliations:** 1 Department of Neurology, Children`s hospital of Fudan University, National Children`s Medical Center, 399 Wanyuan Road, Shanghai, 201102 China; 2 Department of Rehabilitation, Children`s hospital of Fudan University, National Children`s Medical Center, Shanghai, China

**Keywords:** Spinal muscular atrophy (SMA), Hypoplasia of the corpus callosum, Dysmorphism, Nusinersen

## Abstract

**Background:**

Spinal Muscular Atrophy (SMA) is a severe neuromuscular disorder due to a defect in the survival motor neuron 1 (*SMN1*) gene. Hypoplasia of the corpus callosum is underdevelopment or thinness of the corpus callosum. SMA and callosal hypoplasia are relatively rare, and there is limited information sharing the diagnosis and treatment for SMA patients with callosal hypoplasia.

**Case description:**

A boy with callosal hypoplasia, small penis, and small testes had been perceived with motor regression at 5 months. He was referred to the rehabilitation department and neurology department at 7 months. Physical examination showed absent deep tendon reflexes, proximal weakness and significant hypotonia. He was recommended to perform trio whole-exome sequencing (WES) and array comparative genomic hybridization (aCGH) for his complicated conditions. The subsequent nerve conduction study revealed some characteristics of motor neuron diseases. We identified a homozygous deletion in exon 7 of the *SMN1* gene by multiplex ligation-dependent probe amplification and failed to find further pathogenic variations responsible for multiple malformations by trio WES and aCGH. He was diagnosed as SMA. Despite some concerns, he received the therapy of nusinersen for nearly 2 years. He gained the milestone of sitting without support, which he had never accomplished, after the seventh injection, and he continued to improve. During follow-up, there were no adverse events reported and no signs of hydrocephalus.

**Conclusions:**

Some extra features which could not belong to neuromuscular manifestation made the diagnosis and treatment of SMA more complicated.

## Introduction

Spinal muscular atrophy (SMA) is a neurodegenerative disease of the lower motor neurons from the spinal cord and motor nuclei of the brainstem, with progressive muscle atrophy weakness and paralysis. The most common form of SMA is caused by deletions or disease-causing variants in the survival motor neuron 1 (*SMN1*) gene, localized to 5q11.2-q13.3 which segregates as an autosomal recessive trait [1]. The major pathogenic variants identified in SMA patients are the deletion of exon 7 and 8 of the *SMN1* gene or, in some cases, only of exon 7 [2–5]. The incidence is approximately 1 in 10,000 live births [6]. According to the onset age and motor function differences in children with SMA, there are five subtypes of SMA: type I, type II, type III, type IV, and type 0, which refers to the onset of SMA at prenatal period that usually causes death within one month if untreated [6].

The corpus callosum is the major junction between the cerebral hemispheres, extending from the frontal lobe anteriorly to above the quadrigeminal plate posteriorly. Among callosal abnormalities, agenesis of the corpus callosum, which can be partial or complete, and callosal hypoplasia (underdevelopment or thinness) are included [7]. An incidence of callosal abnormalities is difficult to estimate as many isolated cases are asymptomatic. Based on existing data, the prevalence of agenesis and hypoplasia of the corpus callosum in population-based surveys in California and southeastern Hungary was only 1.8 per 10,000 and 2.05 per 10,000 live births, respectively [8, 9]. Abnormalities of the corpus callosum were evident in some chromosomal diseases, indicating that genes may be responsible for the malformation [10, 11]. Association of SMA with hypoplasia of the corpus callosum has rarely been reported.

Here we reported on a SMA boy with hypoplasia of the corpus callosum, some dysmorphic features including small penis and small testes, and low growth. He was finally diagnosed as SMA with 5 months’ delay. Despite some concerns, he started to receive nusinersen at 18-month-old. The purpose of this study was to share the diagnostic experience in this complicated SMA.

## Case presentation

The patient is a 42-month-old boy from a family without ancestral history of neuromuscular disorders. The parents are healthy and non consanguineous. The fetus was found with hypoplasia of the corpus callosum and reduced cerebellum size in the second trimester. There were no antenatal signs for muscle disorders such as polyhydramnios, reduced fetal movements, and no history of hypoxic/ischemic events during pregnancy.

**Fig. 1:**
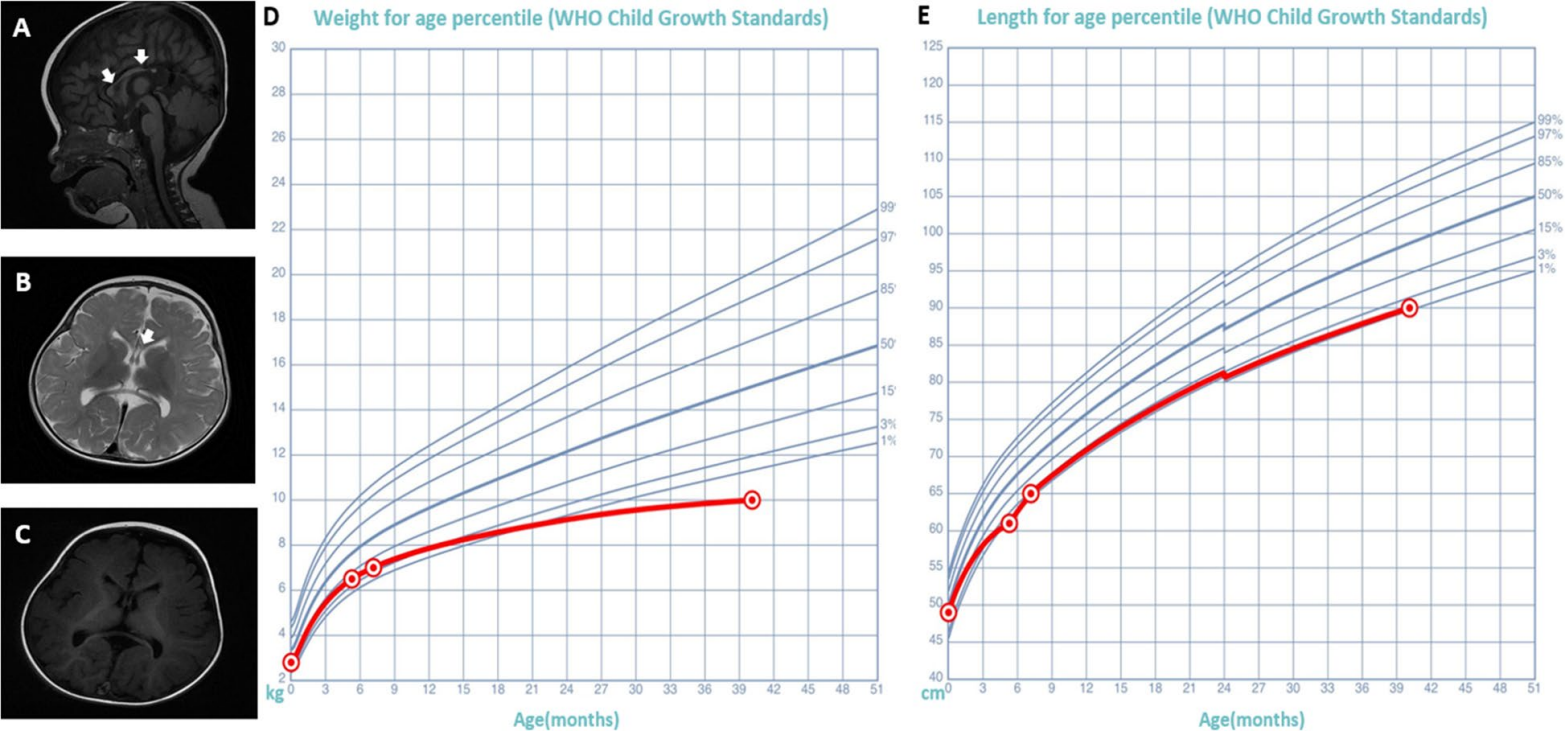
Brain MRI of the patient at 7 months old and the growth curve. Brain MRI showed hypoplasia of the corpus callosum, mainly in the trunk (arrow in A and B) with normal cerebral hemispheres, including the cerebellum and brainstem. **A** slight enlargement in the bilateral ventricles was observed (**B** and **C**). The growth curve showed low growth of the boy (**D** and **E**)

He was born at 39 weeks by uncomplicated Caesarean, with birth weight 2.8 kg and length of 49 cm. The baby started to hold his head up at the age of 3 months and roll over at the age of 4 months. At 5 months he was found with low growth, with body weight 6.5 kg and length of 61 cm (Fig. 1), and abnormal genitals with small penis about 0.5 cm during a routine check-up. Then the patient was referred to endocrinology and urology surgery department of our hospital, respectively. At the initial visit, he was also noted with dysmorphic features including ocular hypertelorism, esotropia and nose-bridge collapse. He was admitted to endocrinology department for further evaluation at the age of 7 months. But the boy had more manifestation beyond those dysmorphic features. When he was admitted to endocrinology department, his parents told the doctors that the boy seemed to start to have motor regression at 5 months of age, manifesting unsteady head control and less movement of the bilateral lower limbs in that admission. Then he was referred to the rehabilitation department and the neurology department after discharged. Examination showed head circumference of 40 cm and proximal leg weakness of 3 and arm weakness of 3 + on medical research council scale (MRC). Deep tendon reflexes were absent and hypotonia was significant.

Lab test showed normal thyroid function and tandem mass spectrometry. Stimulation tests of adrenocorticotrophic hormone, luteinizing hormone-releasing hormone and human chorionic gonadotrophin showed good response. Ultrasound showed small testes, which were 7.9*3.6*4.1 mm of the left one and 8.7*3.3*3.9 mm of the right one. The cranial MRI confirmed hypoplasia of the corpus callosum and normal size of the pituitary and cerebellum (Fig. 1). The subsequent nerve conduction study (NCS) showed reduced compound muscle action potential (CMAP) of the right median and tibial nerves, and needle electromyography showed giant motor unit potentials in all the tested muscles.

The androgen receptor gene (AR) is normal, and there is no pathogenic variation in the steroid 5-alpha-reductase-2 gene (SRD5A2).Considering the boy had hypoplasia of the corpus callosum, facial dysmorphism, unexplained small penis and testes and motor development regression, he was recommended to perform trio whole-exome sequencing (WES) and array comparative genomic hybridization (aCGH) in the rehabilitation department. The SMN multiplex ligation-dependent probe amplification in light of the findings in NCS identified a homozygous deletion in exon 7 of the *SMN1* gene and 3 copies of the *SMN2* gene. No further pathogenic variations could explain the hypoplasia of the corpus callosum and dysmorphic features through trio WES and aCGH. The boy was diagnosed as SMA type I, according to the age at onset and the best motor milestone.

Nusinersen for the boy was discussed after it had been available in China. He might get some benefits from nusinersen, but the symptoms potentially caused by brain malformation are still a big problem for him. After an in-depth discussion, the plan of a therapy of nusinersen was made and we would weigh the pros and cons to reconsider nusinersen after one year.

**Fig. 2:**
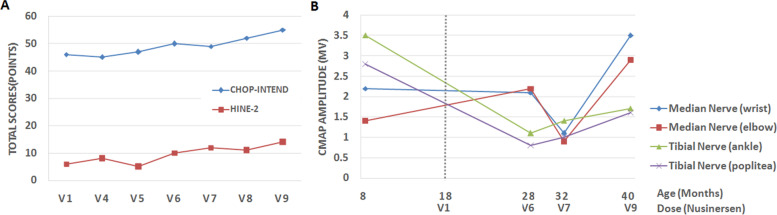
Changes in total scores of the Children’s Hospital of Philadelphia Infant Test of Neuromuscular Disorders (CHOP-INTEND) and the Hammersmith Infant Neurological Examination -2 (HINE-2) from baseline, and changes in compound muscle action potential (CMAP) amplitude. **A**, Changes in the total scores of CHOP-INTEND (the blue line) and HINE-2(the red line) from baseline at 18 months old to 40 months old. **B**, Changes in CMAP amplitude from baseline at 18 months old to 40 months old. The dashed black line denotes he started to receive nusinersen

The Children’s Hospital of Philadelphia Infant Test of Neuromuscular Disorders (CHOP-INTEND) was 46 points, and the Hammersmith Infant Neurological Examination (HINE) Sect. 2 was 6 at the age of 18 months when he started to receive nusinersen and routine habilitation training. As we expected he had made an improvement in motor function after one year, and he continued to complete 9 intrathecal doses as protocol [12]. Physical examination showed proximal leg weakness of MRC grade 3 + and arm weakness of MRC grade 4 at the age of 40 months, and the results of CHOP-INTEND and HINE-2 improved a lot (Fig. 2), as the scores at last evaluation were 55 and 14 points, respectively.

After starting the nusinersen treatment, both the CMAP of tibial nerve and median nerve still declined until the sixth or seventh dose of nusinersen. Notably, the CMAPs started to improve at around 1 year after starting the nusinersen treatment, The changes in the median nerves were more pronounced than the tibial nerves (Fig. 2).

His parents also reported that he regained the ability of rolling over without assistance after the fifth injection. Moreover, he was able to sit independently for several minutes after the seventh injection. He could sit more steadily, lasting for 20 to 30 min at last follow-up.

During therapy, he had an infection of the upper-respiratory tract. No other adverse effects were reported. There were no clinically significant adverse events in laboratory values or physical examinations considered related to the nusinersen treatment.

Although he could speak at the age of 12 months, the progress of speech had been very slow prior to 18-month-old. The speech improved a lot after the age of 30 months, and he could communicate easily. At present, he could speak short sentences with 7 to 8 words. However, his growth was still slow with body weight of 10 kg, body length 90.0 cm (Fig. 1) and head circumference of 48 cm.

At present, his parents are pleased with the improvements in their son and would like to continue the treatment to see if there will be greater motor improvement in the future.

## Discussion and conclusions

Complicated SMA cases deserve more attention with earlier diagnosis and better prognosis in the era of disease-modifying therapy. What did we learn from the experience? Firstly, although hypotonia is not very specific in SMA, when the parents looked back to the development history, the baby might have had slight hypotonia at the age of 2 to 3 months old, which had not caught attention of caregivers and even physicians until motor regression occurred. Secondly, hypoplasia corpus callosum might cause some interference in diagnosis of SMA. Proximal weakness and absent deep tendon reflex, which are very typical in SMA, could not be explained by hypoplasia of corpus callosum. He regressed on motor function while cases with callosal hypoplasia usually have development delay without progression. Finally, the key features of SMA including hypotonia, proximal symmetric weakness and areflexia should be kept in mind and prompt the diagnosis.

The clinical classification in the boy is complicated. The boy seemed to have motor regression about 5 months old and he could not sit independently until 18 months, so he was classified as SMA I. But it is difficult to eliminate the possible effect of callosal hypoplasia on the development in the boy, although callosal hypoplasia might be asymptomatic in some cases. Most patients with SMA I had the scores less than 40 points on CHOP-INTEND [13], however his score on CHOP-INTEND was 46 points by 18 months old before nusinersen therapy.

The case emphasizes the probable association between SMA and callosal hypoplasia and some dysmorphic features. To date, there are a few cases with SMA with having brain malformation, including dysplasia of the corpus callosum in SMA type II as well as supratentorial atrophy, widening of sulcus, and ventricles in SMA type 0 [14, 15]. Although brain involvement in SMA is still unusual, the underlying pathophysiology should be uncovered. SMA is a multisystem disease with dysfunction in peripheral tissues and organs [16], while small penis and small testes has never been reported.

The efficacy and safety of nusinersen in SMA have been shown in the clinical trials [12, 17–19]. In the real world, there are some complicated cases, like the case in our study. The culprits behind of the symptoms are difficult to determine. The questions needing to be answered are the effects of nusinersen and the safety concerns in those complicated SMA cases. As hoped, the motor function improved gradually and continuously, concomitant with the changes of CHOP-INTEND and HINE-2. He gained the very important milestone of sitting without support. But we should attribute the improvement to nusinersen conservatively and cautiously. During his follow-up, there were no adverse events reported and no signs of hydrocephalus. Hydrocephalus has been rarely reported in patients treated with nusinersen [20] or onasemnogene abeparvovec [21]. The incidence rate ratio of hydrocephalus was 4.7 (95% CI: 2.4–10.2) among SMA cases based on US electronic health records, the data before the approval of nusinersen suggests that the SMA disease state may increase the risk of hydrocephalus [22]. Further observation during the nusinersen therapy is needed on the signs of hydrocephalus.

There was apparent delay in enhancement of CMAP amplitude compared with clinical improvement in the boy. Longitudinal changes of CMAP amplitude have been demonstrated some fluctuations over follow-up lasting longer than 3 years in children with SMA type II and type III, while all results of other scales, including the Hammersmith Functional Motor Scale–Expanded, Upper Limb Module test, and 6-Minute Walk Test showed progressive improvements [18]. A similar trend also has been shown in SMA type I [12]. Comparing these above studies, there were some limitations in our study due to deficiency of the data at baseline, the fifth and eighth injection. CMAP as a possible predictive biomarker still needs more evidences.

## Conclusions

So far, the association between the development of the brain, such as the corpus callosum, and SMA reminds us that there are some complicated SMAs. Some extra features which could not belong to neuromuscular manifestation made the diagnosis and treatment more complicated.

## Data Availability

The datasets generated during and/or analyzed during the current study are available from the corresponding author on reasonable request.

## Abbreviations

SMA: Spinal muscular atrophy
SMN1: Survival motor neuron 1
CMAP: Compound muscle action potential
CHOP INTEND: The Children’s Hospital of Philadelphia Infant Test of Neuromuscular Disorders
HINE: The Hammersmith Infant Neurological Examination
